# *N*-(4-Chloro­phen­yl)-3-nitro­pyridin-2-amine

**DOI:** 10.1107/S1600536811045570

**Published:** 2011-11-05

**Authors:** Aina Mardia Akhmad Aznan, Zainal Abidin Fairuz, Zanariah Abdullah, Seik Weng Ng, Edward R. T. Tiekink

**Affiliations:** a Department of Chemistry, University of Malaya, 50603 Kuala Lumpur, Malaysia; b Chemistry Department, Faculty of Science, King Abdulaziz University, PO Box 80203 Jeddah, Saudi Arabia

## Abstract

In the title compound, C_11_H_8_ClN_3_O_2_, the presence of intra­molecular N—H⋯O and C—H⋯N inter­actions help to establish an almost planar mol­ecule [dihedral angle between the pyridine and benzene rings = 9.89 (8)° and r.m.s. deviation for all 17 non-H atoms = 0.120 Å]. Supra­molecular tapes feature in the crystal packing whereby dimeric aggregates sustained by pairs of C—H⋯O inter­actions are connected by π–π inter­actions occurring between translationally related pyridine rings and between translationally related benzene rings along the *b* axis [centroid–centroid distance = length of *b* axis = 3.8032 (4) Å].

## Related literature

For the structure of a related pyrimidine amine derivative, see: Aznan Akhmad *et al.* (2010 ▶).
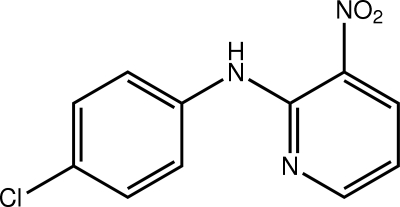


## Experimental

### 

#### Crystal data

C_11_H_8_ClN_3_O_2_
*M* *_r_* = 249.65
Monoclinic, 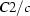

*a* = 30.472 (3) Å
*b* = 3.8032 (4) Å
*c* = 21.300 (2) Å
β = 123.153 (1)°
*V* = 2066.7 (4) Å^3^
*Z* = 8
Mo *K*α radiation
μ = 0.36 mm^−1^
*T* = 100 K
0.40 × 0.15 × 0.05 mm

#### Data collection

Bruker SMART APEX CCD diffractometer
Absorption correction: multi-scan (*SADABS*; Sheldrick, 1996 ▶) *T*_min_ = 0.869, *T*_max_ = 0.982
8919 measured reflections
2347 independent reflections
1912 reflections with *I* > 2σ(*I*)
*R*_int_ = 0.042

#### Refinement

*R*[*F* ^2^ > 2σ(*F* ^2^)] = 0.034
*wR*(*F* ^2^) = 0.091
*S* = 1.00
2347 reflections
158 parameters
H atoms treated by a mixture of independent and constrained refinement
Δρ_max_ = 0.33 e Å^−3^
Δρ_min_ = −0.22 e Å^−3^

### 

Data collection: *APEX2* (Bruker, 2009 ▶); cell refinement: *SAINT* (Bruker, 2009 ▶); data reduction: *SAINT*; program(s) used to solve structure: *SHELXS97* (Sheldrick, 2008 ▶); program(s) used to refine structure: *SHELXL97* (Sheldrick, 2008 ▶); molecular graphics: *ORTEP-3* (Farrugia, 1997 ▶) and *DIAMOND* (Brandenburg, 2006 ▶); software used to prepare material for publication: *publCIF* (Westrip, 2010 ▶).

## Supplementary Material

Crystal structure: contains datablock(s) global, I. DOI: 10.1107/S1600536811045570/hb6473sup1.cif

Structure factors: contains datablock(s) I. DOI: 10.1107/S1600536811045570/hb6473Isup2.hkl

Supplementary material file. DOI: 10.1107/S1600536811045570/hb6473Isup3.cml

Additional supplementary materials:  crystallographic information; 3D view; checkCIF report

## Figures and Tables

**Table 1:** Hydrogen-bond geometry (Å, °)

*D*—H⋯*A*	*D*—H	H⋯*A*	*D*⋯*A*	*D*—H⋯*A*
N1—H1*n*⋯O1	0.87 (2)	1.91 (2)	2.6280 (18)	138.2 (17)
C7—H7⋯N2	0.95	2.31	2.909 (2)	120
C3—H3⋯O2^i^	0.95	2.48	3.340 (3)	152

Symmetry code: (i) 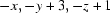.

**Fig. 1.**
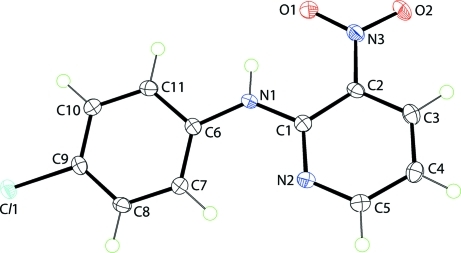
The molecular structure of (I) showing displacement ellipsoids at the 50% probability level.

**Fig. 2.**
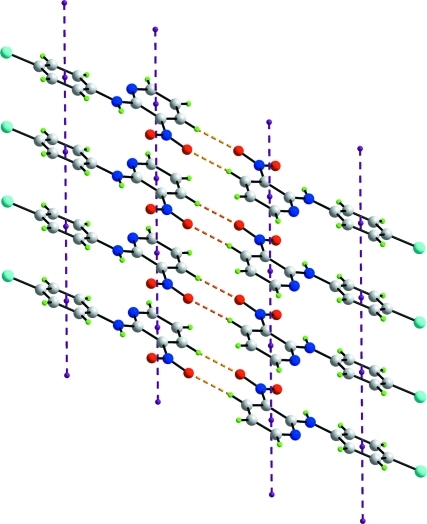
Supramolecular tape along the b axis in (I) sustained by C—H···O and π–π interactions shown as orange and purple dashed lines, respectively.

**Fig. 3.**
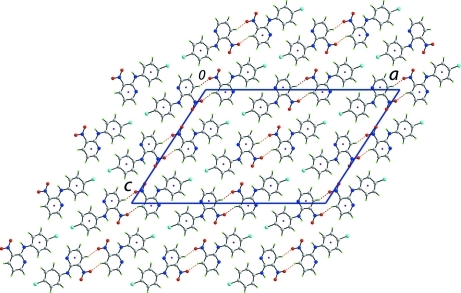
Unit-cell contents for (I) shown in projection down the b axis. The C—H···O interactions are shown as orange dashed lines.

## supplementary crystallographic information

### Comment

In connection with synthetic and structural studies of nitro-pyridine/pyrimidine derivatives (Aznan Akhmad *et al.*, 2010), the title compound, (I), was investigated. A small twist is evident in (I), Fig. 1, as seen in the value of the dihedral angle between the pyridyl and benzene rings of 9.89 (8) Å. The nitro group is co-planar with the pyridyl ring to which it is connected: the O1—N3—C2—C1 torsion angle is 5.0 (2)°. The observed conformation is stabilized by an intramolecular N—H···O hydrogen bond as well as a C—H···N interaction, Table 1. Overall, the molecule is close to planar with the r.m.s. deviation for all 17 non-hydrogen atoms being 0.120 Å.

In the crystal structure, centrosymmetrically related molecules are connected into dimeric aggregates *via* C—H···O interactions, Table 1. These are connected into a supramolecular tape along the *b* axis by π–π interactions between translationally related pyridyl rings and between translationally related benzene rings with the centroid···centroid separation corresponding to the length of the *b* axis, *i.e*. 3.8032 (4) Å, Fig. 2. The columns are connected by weak C—H···Cl [closest contact: C5—H5···Cl1^i^ = 3.97 Å, C5···Cl1^i^ = 3.6112 (19) Å and angle at H5 = 124° for *i*: 1/2 - *x*, 3/2 - *y*, 1 - *z*] and Cl···Cl [Cl1···Cl1^ii^ = 3.4366 (7) Å for *ii*: 1/2 - *x*, -1/2 + *y*, 1/2 - *z*] contacts. A view of the unit-cell contents is given in Fig. 3.

### Experimental

2-Chloro-3-nitro-pyridine (0.906 g, 0.0057 mol) and *p*-chloroaniline (0.730 g, 0.0057 mol) were refluxed in ethanol (5 ml) for 4 h at 385 K. After cooling the mixture, the residue was dissolved in a minimum volume of water (10 ml) and extracted with ether (3 *x* 10 ml). The ethereal layer was washed with water and dried over anhydrous sodium sulfate. Evaporation gave a red solid and recrystallization from its diethyl ether solution yielded red-brown prisms of (I) after a few days.

### Refinement

Carbon-bound hydrogen atoms were placed at calculated positions (C—H 0.95 Å) and were treated as riding on their parent carbon atoms, with *U*(H) set to 1.2 times *U*_eq_(C). The amine-H atom was refined with N—H = 0.86±0.01 Å with refined *U*_iso_.

### Crystal data

**Table:**

C_11_H_8_ClN_3_O_2_	*F*(000) = 1024
*M**_r_* = 249.65	*D*_x_ = 1.605 Mg m^−^^3^
Monoclinic, *C*2/*c*	Mo *K*α radiation, λ = 0.71073 Å
Hall symbol: -C 2yc	Cell parameters from 2586 reflections
*a* = 30.472 (3) Å	θ = 2.3–28.0°
*b* = 3.8032 (4) Å	µ = 0.36 mm^−^^1^
*c* = 21.300 (2) Å	*T* = 100 K
β = 123.153 (1)°	Prism, red-brown
*V* = 2066.7 (4) Å^3^	0.40 × 0.15 × 0.05 mm
*Z* = 8	

### Data collection

**Table:**

Bruker SMART APEX CCD diffractometer	2347 independent reflections
Radiation source: fine-focus sealed tube	1912 reflections with *I* > 2σ(*I*)
graphite	*R*_int_ = 0.042
ω scans	θ_max_ = 27.5°, θ_min_ = 1.6°
Absorption correction: multi-scan (*SADABS*; Sheldrick, 1996)	*h* = −36→38
*T*_min_ = 0.869, *T*_max_ = 0.982	*k* = −4→4
8919 measured reflections	*l* = −27→27

### Refinement

**Table:**

Refinement on *F*^2^	Primary atom site location: structure-invariant direct methods
Least-squares matrix: full	Secondary atom site location: difference Fourier map
*R*[*F*^2^ > 2σ(*F*^2^)] = 0.034	Hydrogen site location: inferred from neighbouring sites
*wR*(*F*^2^) = 0.091	H atoms treated by a mixture of independent and constrained refinement
*S* = 1.00	*w* = 1/[σ^2^(*F*_o_^2^) + (0.0483*P*)^2^ + 1.1995*P*] where *P* = (*F*_o_^2^ + 2*F*_c_^2^)/3
2347 reflections	(Δ/σ)_max_ = 0.001
158 parameters	Δρ_max_ = 0.33 e Å^−^^3^
0 restraints	Δρ_min_ = −0.22 e Å^−^^3^

### Special details

**Table:**

Refinement. Refinement of *F*^2^ against ALL reflections. The weighted *R*-factor *wR* and goodness of fit *S* are based on *F*^2^, conventional *R*-factors *R* are based on *F*, with *F* set to zero for negative *F*^2^. The threshold expression of *F*^2^ > 2σ(*F*^2^) is used only for calculating *R*-factors(gt) *etc*. and is not relevant to the choice of reflections for refinement. *R*-factors based on *F*^2^ are statistically about twice as large as those based on *F*, and *R*- factors based on ALL data will be even larger.

### Fractional atomic coordinates and isotropic or equivalent isotropic displacement parameters (Å^2^)

**Table:**

	*x*	*y*	*z*	*U*_iso_*/*U*_eq_	
Cl1	0.223442 (15)	0.15167 (11)	0.28841 (2)	0.01760 (13)	
O1	0.00287 (5)	1.1095 (3)	0.32850 (6)	0.0239 (3)	
O2	−0.01657 (5)	1.3711 (4)	0.40019 (7)	0.0271 (3)	
N1	0.09477 (5)	0.8069 (4)	0.38672 (8)	0.0151 (3)	
H1n	0.0640 (8)	0.866 (5)	0.3481 (11)	0.021 (5)*	
N2	0.14771 (5)	0.8205 (4)	0.51652 (7)	0.0155 (3)	
N3	0.01356 (5)	1.1930 (4)	0.39171 (8)	0.0180 (3)	
C1	0.10203 (6)	0.9037 (4)	0.45291 (9)	0.0141 (3)	
C2	0.06265 (6)	1.0832 (4)	0.45711 (9)	0.0148 (3)	
C3	0.07133 (7)	1.1672 (4)	0.52649 (9)	0.0175 (3)	
H3	0.0452	1.2859	0.5296	0.021*	
C4	0.11814 (7)	1.0768 (4)	0.59046 (9)	0.0181 (4)	
H4	0.1252	1.1299	0.6388	0.022*	
C5	0.15475 (6)	0.9047 (4)	0.58188 (9)	0.0172 (3)	
H5	0.1872	0.8427	0.6261	0.021*	
C6	0.12870 (6)	0.6443 (4)	0.36965 (9)	0.0137 (3)	
C7	0.18163 (6)	0.5712 (4)	0.42051 (9)	0.0161 (3)	
H7	0.1978	0.6249	0.4722	0.019*	
C8	0.21055 (6)	0.4195 (4)	0.39510 (9)	0.0163 (3)	
H8	0.2467	0.3708	0.4294	0.020*	
C9	0.18699 (6)	0.3393 (4)	0.32026 (9)	0.0149 (3)	
C10	0.13421 (6)	0.4065 (4)	0.26931 (9)	0.0161 (3)	
H10	0.1181	0.3478	0.2179	0.019*	
C11	0.10550 (6)	0.5593 (4)	0.29426 (9)	0.0158 (3)	
H11	0.0694	0.6076	0.2596	0.019*	

### Atomic displacement parameters (Å^2^)

**Table:**

	*U*^11^	*U*^22^	*U*^33^	*U*^12^	*U*^13^	*U*^23^
Cl1	0.0176 (2)	0.0184 (2)	0.0213 (2)	0.00078 (16)	0.01348 (17)	−0.00104 (16)
O1	0.0167 (6)	0.0340 (8)	0.0164 (6)	0.0051 (5)	0.0062 (5)	−0.0024 (5)
O2	0.0182 (6)	0.0350 (8)	0.0279 (7)	0.0118 (6)	0.0125 (5)	−0.0006 (6)
N1	0.0103 (7)	0.0182 (7)	0.0148 (6)	0.0020 (6)	0.0056 (6)	−0.0004 (6)
N2	0.0130 (7)	0.0168 (7)	0.0157 (6)	0.0005 (6)	0.0071 (6)	0.0011 (5)
N3	0.0148 (7)	0.0186 (7)	0.0213 (7)	0.0018 (6)	0.0103 (6)	−0.0002 (6)
C1	0.0148 (8)	0.0107 (8)	0.0176 (7)	−0.0025 (6)	0.0094 (6)	−0.0004 (6)
C2	0.0115 (8)	0.0141 (8)	0.0184 (8)	−0.0007 (6)	0.0079 (6)	−0.0003 (6)
C3	0.0197 (8)	0.0143 (8)	0.0234 (8)	0.0004 (7)	0.0148 (7)	0.0006 (6)
C4	0.0225 (9)	0.0167 (8)	0.0178 (8)	−0.0018 (7)	0.0128 (7)	−0.0008 (6)
C5	0.0157 (8)	0.0178 (9)	0.0161 (7)	−0.0009 (7)	0.0073 (6)	0.0013 (6)
C6	0.0145 (8)	0.0104 (8)	0.0178 (7)	−0.0013 (6)	0.0098 (6)	0.0000 (6)
C7	0.0157 (8)	0.0170 (8)	0.0152 (7)	−0.0004 (7)	0.0082 (7)	−0.0016 (6)
C8	0.0115 (8)	0.0174 (8)	0.0183 (8)	0.0000 (6)	0.0070 (6)	−0.0001 (6)
C9	0.0168 (8)	0.0114 (8)	0.0206 (8)	0.0000 (6)	0.0129 (7)	0.0004 (6)
C10	0.0163 (8)	0.0158 (8)	0.0149 (7)	−0.0015 (6)	0.0078 (6)	−0.0006 (6)
C11	0.0129 (8)	0.0148 (8)	0.0169 (7)	−0.0004 (6)	0.0065 (6)	0.0003 (6)

### Geometric parameters (Å, °)

**Table:**

Cl1—C9	1.7400 (16)	C4—C5	1.389 (2)
O1—N3	1.2408 (18)	C4—H4	0.9500
O2—N3	1.2312 (18)	C5—H5	0.9500
N1—C1	1.353 (2)	C6—C11	1.393 (2)
N1—C6	1.412 (2)	C6—C7	1.393 (2)
N1—H1n	0.87 (2)	C7—C8	1.387 (2)
N2—C5	1.327 (2)	C7—H7	0.9500
N2—C1	1.346 (2)	C8—C9	1.378 (2)
N3—C2	1.440 (2)	C8—H8	0.9500
C1—C2	1.426 (2)	C9—C10	1.386 (2)
C2—C3	1.389 (2)	C10—C11	1.376 (2)
C3—C4	1.372 (2)	C10—H10	0.9500
C3—H3	0.9500	C11—H11	0.9500
			
C1—N1—C6	131.43 (14)	N2—C5—H5	117.6
C1—N1—H1n	113.1 (12)	C4—C5—H5	117.6
C6—N1—H1n	115.4 (12)	C11—C6—C7	119.45 (15)
C5—N2—C1	118.99 (14)	C11—C6—N1	114.64 (14)
O2—N3—O1	121.71 (14)	C7—C6—N1	125.90 (14)
O2—N3—C2	118.75 (13)	C8—C7—C6	119.47 (14)
O1—N3—C2	119.54 (13)	C8—C7—H7	120.3
N2—C1—N1	118.36 (14)	C6—C7—H7	120.3
N2—C1—C2	119.48 (14)	C9—C8—C7	120.22 (15)
N1—C1—C2	122.15 (14)	C9—C8—H8	119.9
C3—C2—C1	120.01 (15)	C7—C8—H8	119.9
C3—C2—N3	117.09 (14)	C8—C9—C10	120.84 (15)
C1—C2—N3	122.88 (14)	C8—C9—Cl1	120.15 (13)
C4—C3—C2	119.25 (15)	C10—C9—Cl1	119.02 (12)
C4—C3—H3	120.4	C11—C10—C9	119.08 (15)
C2—C3—H3	120.4	C11—C10—H10	120.5
C3—C4—C5	117.45 (15)	C9—C10—H10	120.5
C3—C4—H4	121.3	C10—C11—C6	120.93 (15)
C5—C4—H4	121.3	C10—C11—H11	119.5
N2—C5—C4	124.83 (15)	C6—C11—H11	119.5
			
C5—N2—C1—N1	−178.05 (15)	C1—N2—C5—C4	−0.2 (3)
C5—N2—C1—C2	0.7 (2)	C3—C4—C5—N2	−0.2 (3)
C6—N1—C1—N2	−4.5 (3)	C1—N1—C6—C11	175.03 (16)
C6—N1—C1—C2	176.74 (16)	C1—N1—C6—C7	−6.0 (3)
N2—C1—C2—C3	−0.7 (2)	C11—C6—C7—C8	0.9 (2)
N1—C1—C2—C3	177.97 (16)	N1—C6—C7—C8	−178.01 (15)
N2—C1—C2—N3	177.63 (14)	C6—C7—C8—C9	−0.5 (3)
N1—C1—C2—N3	−3.7 (3)	C7—C8—C9—C10	−0.3 (3)
O2—N3—C2—C3	4.2 (2)	C7—C8—C9—Cl1	179.57 (13)
O1—N3—C2—C3	−176.53 (15)	C8—C9—C10—C11	0.8 (2)
O2—N3—C2—C1	−174.27 (15)	Cl1—C9—C10—C11	−179.10 (13)
O1—N3—C2—C1	5.0 (2)	C9—C10—C11—C6	−0.4 (3)
C1—C2—C3—C4	0.3 (3)	C7—C6—C11—C10	−0.4 (2)
N3—C2—C3—C4	−178.19 (15)	N1—C6—C11—C10	178.60 (15)
C2—C3—C4—C5	0.2 (3)		

### Hydrogen-bond geometry (Å, °)

**Table:**

*D*—H···*A*	*D*—H	H···*A*	*D*···*A*	*D*—H···*A*
N1—H1n···O1	0.87 (2)	1.91 (2)	2.6280 (18)	138.2 (17)
C7—H7···N2	0.95	2.31	2.909 (2)	120
C3—H3···O2^i^	0.95	2.48	3.340 (3)	152

Symmetry codes: (i) −*x*, −*y*+3, −*z*+1.

## References

[bb1] Aznan Akhmad, M. A., Abdullah, Z., Fairuz, Z. A., Ng, S. W. & Tiekink, E. R. T. (2010). *Acta Cryst.* E**66**, o2400. 10.1107/S1600536810033040 PMC3008084 21588732

[bb2] Brandenburg, K. (2006). *DIAMOND* Crystal Impact GbR, Bonn, Germany.

[bb3] Bruker (2009). *APEX2* and *SAINT* Bruker AXS Inc., Madison, Wisconsin, USA.

[bb4] Farrugia, L. J. (1997). *J. Appl. Cryst.* **30**, 565.

[bb5] Sheldrick, G. M. (1996). *SADABS* University of Göttingen, Germany.

[bb6] Sheldrick, G. M. (2008). *Acta Cryst.* A**64**, 112–122. 10.1107/S0108767307043930 18156677

[bb7] Westrip, S. P. (2010). *J. Appl. Cryst.* **43**, 920–925.

